# Effect of Propylene Glycol Coolant pH on the Galvanic Corrosion Behavior of 6061 Aluminum Alloy/304 Stainless Steel

**DOI:** 10.3390/ma19132898

**Published:** 2026-07-06

**Authors:** Hao Miao, Cong Shao, Jinqiao Zheng, Hao Yu, Heqian Wang, Kui Xiao

**Affiliations:** 1 Institute for Advanced Materials and Technology, University of Science and Technology Beijing, Beijing 100083, China; 2 ZTE Corporation, Shenzhen 518057, China; shao.cong@zte.com.cn (C.S.);

**Keywords:** propylene glycol coolant, aluminum alloy, stainless steel, galvanic corrosion, pH value

## Abstract

6061 aluminum alloy is lightweight and has good thermal conductivity, while 304 stainless steel possesses excellent mechanical properties and corrosion resistance; both have broad application prospects in cooling circuits. Propylene glycol coolant shows great potential in liquid cooling systems due to its low toxicity and good antifreeze properties. However, during operation, galvanic corrosion may occur when the two metals come into direct contact within the coolant, thereby threatening system safety and service life. This study focuses on 6061 aluminum alloy, 304 stainless steel, and their galvanic couples. Electrochemical testing, SEM, 3D confocal microscopy, and XPS were used to systematically investigate their self-corrosion and galvanic corrosion behavior in propylene glycol coolant at pH values of 4.8, 6.8, and 8.8. The results indicate that 6061 aluminum alloy is more sensitive to pH changes; its corrosion resistance first increases and then decreases as pH rises, with the least corrosion occurring at pH = 6.8 and the most severe at pH = 4.8. 304 stainless steel exhibited lower corrosion rates at pH 6.8 and 8.8, but corrosion significantly worsened at pH 4.8. For the 6061 aluminum alloy/304 stainless steel couple, the galvanic current first decreased and then increased with rising pH, while the galvanic potential first increased and then decreased. The 6061 aluminum alloy consistently acted as the anode, and the 304 stainless steel consistently acted as the cathode, with the highest sensitivity to galvanic corrosion observed at pH 4.8. XPS analysis shows that under different pH conditions, the corrosion products of 6061 aluminum alloy are Al(OH)_3_ and Al_2_O_3_, while the main components of the passivation film on 304 stainless steel remain unchanged.

## 1. Introduction

With the continuous advancement of liquid cooling technology in the automotive, aerospace, new energy, and data center sectors, coolants are playing an increasingly critical role in ensuring system safety and efficient heat transfer. To meet the demands of high power density and high reliability, coolants must possess excellent heat transfer properties, freeze resistance, thermal stability, material compatibility, and long-term operational stability [1,2,3,4]. In actual liquid cooling systems, components such as cold plates, piping, fittings, valve bodies, manifolds, and heat exchangers are often composed of different metallic materials. Among these, aluminum alloys are lightweight and have excellent thermal conductivity, making them commonly used for cold plates, heat dissipation structures, and heat exchange components; stainless steel, with its high mechanical strength and corrosion resistance, is frequently used for piping, fittings, valve bodies, and connecting structures [5]. Aluminum alloys and stainless steel are frequently used in combination within cooling circuits; however, direct contact between the two may trigger galvanic corrosion [6]. Consequently, aluminum alloys and stainless steel are often paired in cooling systems based on the specific functions of different components, and their material compatibility significantly influences the stable operation of the cooling system.

Currently, the mainstream coolants in engineering applications are ethylene glycol/water and propylene glycol/water systems, both of which can adjust freezing and boiling points to meet the requirements of operation across a wide temperature range [7,8]. Ethylene glycol-based coolants have low viscosity and high heat transfer efficiency, but they are prone to oxidation at high temperatures, producing acidic byproducts such as glycolic acid and oxalic acid, which cause a drop in pH. This can damage the passivation films on the surfaces of aluminum alloys and stainless steel, inducing failure modes such as pitting corrosion and crevice corrosion [9]. Although pure ethylene glycol can inhibit the anodic dissolution of aluminum alloys and form protective aluminum-alcohol products, its oxidation products still significantly exacerbate corrosion under long-term high-temperature service conditions [10,11,12]. For stainless steel, the acidic medium generated by the oxidation of ethylene glycol weakens the density of the passivation film, increasing the risk of pitting corrosion [13,14,15]. In contrast, propylene glycol-based coolants are less toxic and offer better environmental compatibility and operational safety [16,17], demonstrating greater application potential in scenarios such as data center cold plates [18,19] and new energy vehicle cooling systems [20]. Studies indicate that propylene glycol can inhibit the corrosion of aluminum alloys. This effect is not limited to physical adsorption: the hydroxyl groups in propylene glycol may interact with Al–OH or Al^3+^ sites on the aluminum surface, promoting the formation of Al–O–C-containing alcoholate/organic-oxide interfacial species. Such a layer can reduce the direct contact between water, dissolved oxygen, aggressive ions, and the aluminum substrate, thereby slowing charge transfer and anodic dissolution. However, this protective effect may weaken under acidic or alkaline conditions because the aluminum oxide/alcoholate interfacial layer becomes less stable [21,22]. In addition, aluminum alloys remain highly sensitive to temperature, Cl^−^, and pH in glycol-based coolant environments, making them prone to pitting and localized accelerated corrosion [23]. Stainless steel is generally more stable in similar environments, but when aluminum comes into direct contact with stainless steel and is used in conjunction with conductive organic coolants, there is a risk of galvanic corrosion [24,25,26].

In practical liquid-cooling systems, pH and electrical conductivity are important indicators for evaluating coolant stability and corrosion risk. Recent engineering guidelines for liquid-cooled data centers recommend low-conductivity water for system preparation, with the conductivity controlled below 50–60 μS/cm [27]. For inhibited propylene glycol-based heat transfer fluids, the typical pH is usually maintained in the weakly alkaline range, for example 8.0–10.5, while a decrease in pH below about 7.5 may increase the risk of corrosion and fouling [28]. Therefore, quantitative control of pH and conductivity is necessary for assessing the corrosion behavior of mixed-metal propylene glycol cooling systems. During long-term operation of liquid-cooling systems, oxidation and degradation of the cooling medium or the dissolution of ambient gases can cause changes in the pH of the coolant, which in turn affects the stability of passivation or oxidation layers on metal surfaces and alters the corrosion susceptibility of the materials [29]. Generally speaking, under acidic conditions, surface films are prone to dissolution, leading to accelerated corrosion; under near-neutral conditions, the films are relatively stable; and under alkaline conditions, OH^−^ ions may cause localized damage to the films, resulting in a renewed increase in corrosion susceptibility [30,31]. A study on the corrosion behavior of 7A09 aluminum alloy in propylene glycol coolant under different pH conditions found that at pH = 4, the surface oxide film of the aluminum alloy underwent acid dissolution, resulting in the most severe corrosion; under near-neutral conditions, propylene glycol interacted synergistically with the oxide film to form a stable protective film, significantly reducing the corrosion rate; at pH = 9, OH^−^ caused localized dissolution of the passivation film, leading to a renewed increase in corrosion severity [32]. Research has shown that in ethylene glycol–water solutions, higher pH values enhance the corrosive effect of glycolic acid on AA6061 aluminum alloy [33]. The mechanism involves the formation of complexes between glycolic acid and aluminum ions, which promotes the dissolution of the aluminum matrix [34]. In actual liquid cooling systems, when aluminum alloys come into direct contact with dissimilar metals such as stainless steel, an electrochemical couple is formed in the electrolyte environment of the coolant due to the potential difference between the two, leading to accelerated corrosion of the more active metal (aluminum alloy) [35,36,37,38,39]. The galvanic corrosion behavior of dissimilar-metal couples can be interpreted using mixed potential theory. According to this theory, the galvanic potential is determined by the balance between the anodic dissolution reaction on the active metal and the cathodic reduction reaction on the more noble metal [40]. In addition, galvanic corrosion modeling studies based on polarization curves further show that electrode polarization behavior can be used to predict the galvanic current and potential distribution of coupled metals [41]. A study on the effect of pH on the galvanic corrosion behavior of a three-metal couple consisting of 2024 aluminum alloy, Q235 carbon steel, and 304 stainless steel showed that pH significantly alters the anodic dissolution behavior of the aluminum alloy in the galvanic system [42]. In mixed-metal cooling systems, cations generated by the corrosion of copper or iron alloys deposit on the surface of less active alloys (such as aluminum), forming localized cathodes and significantly increasing the risk of localized corrosion [43].

Overall, previous studies have shown that glycol coolants, pH, and dissimilar-metal contact can affect metal corrosion; however, these factors have mostly been investigated separately. For a 6061 aluminum alloy/304 stainless steel couple in propylene glycol coolant, corrosion is governed not only by the passivation behavior of each metal, but also by the pH-dependent galvanic interaction between anodic aluminum dissolution and cathodic reactions on stainless steel. Nevertheless, the galvanic current evolution and corrosion severity of this couple under acidic, near-neutral, and weakly alkaline conditions remain insufficiently quantified. Therefore, this study investigates the self-corrosion and galvanic corrosion behavior of 6061 aluminum alloy, 304 stainless steel, and their galvanic couples in 25 vol.% propylene glycol coolant at pH 4.8, 6.8, and 8.8. By combining electrochemical measurements, galvanic current monitoring, immersion weight-loss testing, surface morphology observation, and XPS analysis, this work aims to clarify the pH-dependent galvanic corrosion mechanism and provide guidance for coolant pH control in mixed-metal liquid-cooling systems.

## 2. Materials and Methods

### 2.1. Experimental Materials and Coolant

The chemical composition (wt/%) of the 6061 aluminum alloy (T6 condition) used in this study is as follows: 1.08 Mg, 0.68 Si, 0.51 Fe, 0.31 Cu, 0.16 Mn, 0.02 Zn, 0.29 Cr; The chemical composition (wt/%) of 304 stainless steel is: 18.25% Cr, 8.18% Ni, 0.06% C, 1.04% Mn, 0.56% Si, 0.02% S, 0.03% P, and 0.07% Cu. The specimens were machined using wire cutting into two sizes: 10 × 10 × 2 mm and 50 × 25 × 2 mm, for use in electrochemical testing and corrosion immersion testing, respectively. For the corrosion immersion specimens, a small hole with a diameter of 3 mm was drilled 5 mm from the top of the specimen to facilitate suspension. After machining, all specimens were mechanically ground stepwise using SiC abrasive papers from 400 to 2000 grit to remove machining marks and obtain a uniform surface condition. The specimens were then further polished using a 50 nm polishing suspension to obtain a smooth and consistent surface finish. After polishing, the specimens were rinsed with deionized water, ultrasonically cleaned in anhydrous ethanol for 5 min, dried, and prepared for subsequent tests.

The base coolant used in the experiments consists of 75% deionized water and 25% propylene glycol (analytical grade, ≥99.5%) by volume. The pH of the acidic coolant was adjusted using dilute HCl, while that of the alkaline coolant was adjusted using dilute NaOH. This method inevitably introduced additional Cl^−^ and Na^+^ ions and changed the ionic strength of the coolant to some extent. Therefore, the pH effect discussed in this study may also include the influence of ions introduced during pH adjustment, which is acknowledged as a limitation of the present work. Before corrosion testing, the initial electrical conductivity of the prepared 25 vol.% propylene glycol coolant was measured as 33.5 μS/cm at room temperature. The coolant was not deaerated or oxygen-saturated, and all tests were conducted under naturally aerated laboratory conditions.

### 2.2. Coolant Corrosion Test Method

The coolant volume was determined according to GB 29743.2-2025 [44] in which seven 50 × 25 mm metal specimens require 750 mL of coolant. For the self-corrosion tests, the specimens were suspended in a 1000 mL beaker, and the liquid level was maintained approximately 20 mm below the top of the container. For the galvanic corrosion tests, 6061 aluminum alloy and 304 stainless steel were electrically connected using copper wires, and the distance between the two specimens was maintained at 25 mm. To simulate the operating temperature of coolant in data-center liquid-cooling systems, the beakers containing the specimens and coolant were placed in a constant-temperature chamber and maintained at 80 ± 0.5 °C throughout the immersion tests. All specimens were fully immersed in the coolant during the experiment, and the liquid level was kept approximately 20 mm below the top of the beaker to avoid evaporation loss and ensure stable exposure conditions.

### 2.3. Corrosion Testing and Analysis Methods

#### 2.3.1. Corrosion-Induced Weight Loss

Since stainless steel exhibits good corrosion resistance in coolant, this study focuses on the removal of corrosion products from aluminum alloys and the analysis of corrosion weight loss. In accordance with GB/T 16545-2025 [45] concentrated nitric acid (65–68 wt.%, pH < 1) was used to remove corrosion products from the specimen surfaces, which were then weighed (to the nearest 0.01 mg). The corrosion rate (g/m^2^) was calculated based on the weight loss.

#### 2.3.2. Characterization of Corrosion Products

A FEI Quanta 250 scanning electron microscope (SEM, FEI Company, Hillsboro, OR, USA) was used to observe the microscopic corrosion morphology on the sample surface; a KEYENCE VK200 confocal microscope was used to examine the morphology of pitting corrosion pits on the samples after rust removal; A Thermo Fisher Sigma Probe X-ray photoelectron spectrometer (XPS) was used to analyze the chemical states of elements in the surface products of the samples. The test voltage was set to 15 kV, and measurements were performed using monochromatic Al Kα X-rays with a step size of 0.05 eV. The data were then analyzed using Advantage 5.9931 software.

#### 2.3.3. Electrochemical Testing

AC impedance and polarization curve tests were performed using a CS 350H electrochemical workstation with a three-electrode system: working electrodes—6061 aluminum alloy (1 cm^2^) and 304 stainless steel (1 cm^2^); reference electrode—saturated calomel electrode (SCE); and counter electrode—platinum foil (1 × 1 cm). The open-circuit potential (OCP) was stabilized for 30 min. AC impedance spectroscopy was performed at frequencies ranging from 100 kHz to 10 mHz. Polarization scans were conducted at ±500 mV vs. OCP (6061 aluminum alloy) and −500 mV to 2000 mV vs. OCP (304 stainless steel), with a scan rate of 0.5 mV/s. The test solutions used for electrochemical testing were propylene glycol coolants under various environmental conditions.

#### 2.3.4. Galvanic Corrosion Testing and Analysis

A CST 508 multi-channel galvanic corrosion tester was used to measure the galvanic current and potential between 6061 aluminum alloy and 304 stainless steel. The active area of each specimen was 1 cm^2^, and the distance between the 6061 aluminum alloy and the 304 stainless steel was 25 mm. The 6061 aluminum alloy was connected to WE1, the 304 stainless steel to WE2, and SCE to RE. The total test duration was 20 h, comprising 4 h of high-frequency testing, 8 h of medium-frequency testing, and 8 h of low-frequency testing.

The average galvanic current is calculated using the integration method based on the galvanic current-time curve, and the average galvanic current density is then determined based on the actual area of the anode in the galvanic cell. The calculation formula is as follows:

(1) $$i_{g}=\frac{\int_{0}^{t}I_{g}\left(t\right)\mathrm{dt}}{S\;\times \;t}$$

In the equation, I_g_(t) represents the galvanic current at time t (A); S represents the effective working area of the anode specimen (cm^2^); and t represents the test duration (h).

In accordance with HB 5374-1987 [46], the galvanic corrosion susceptibility of 6061 aluminum alloy/304 stainless steel galvanic couples in propylene glycol coolants with three different pH values was classified, and the specific classification results are shown in Table 1.

## 3. Results

### 3.1. Corrosion-Induced Weight Loss

**Figure 1:**
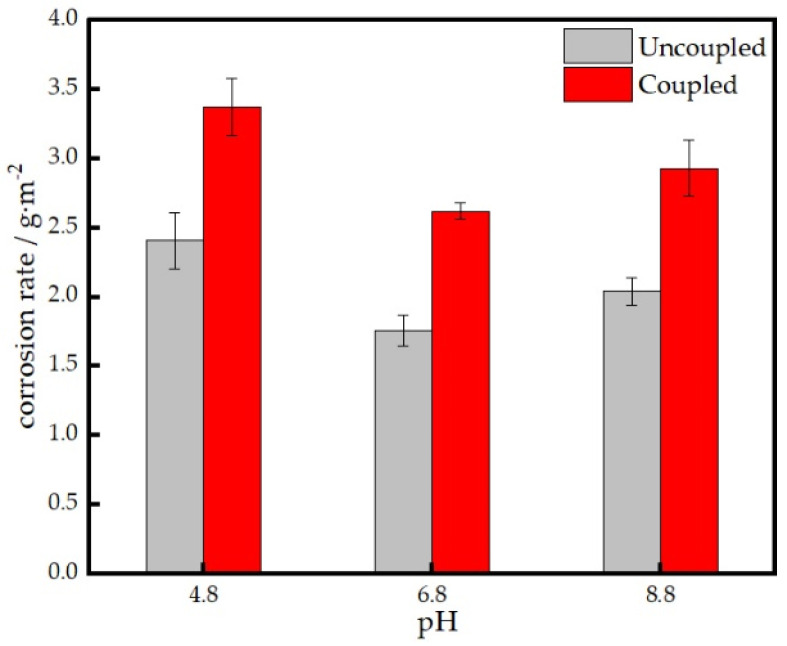
Corrosion rates of 6061 aluminum alloy after immersion in propylene glycol coolant with different pH values for 14 days: uncoupled condition and galvanically coupled with 304 stainless steel.

After immersing 6061 aluminum alloy (single metal) and a 6061/304 galvanic couple in propylene glycol coolant at different pH levels for 14 days, rust was removed from the 6061 aluminum alloy and the weight was measured. The corrosion rates under different test conditions were calculated, as shown in Figure 1. The data are presented with error bars representing the standard deviation of repeated measurements. As shown in Figure 1, corrosion weight loss for both the single metal and the galvanic couple was highest at pH = 4.8, lowest at pH = 6.8, and intermediate at pH = 8.8. Under pH 4.8, 6.8, and 8.8 conditions, the corrosion weight loss of single 6061 aluminum alloy was 2.407, 1.757, and 2.043 g·m^−2^, respectively, while that of 6061 aluminum alloy in the 6061/304 galvanic couple was 3.375, 2.621, and 2.932 g·m^−2^, respectively. Compared with the single-metal state, galvanic coupling increased the corrosion weight loss of 6061 aluminum alloy by 0.968, 0.864, and 0.889 g·m^−2^ at pH 4.8, 6.8, and 8.8, respectively. This indicates that galvanic coupling with 304 stainless steel promoted the anodic dissolution of 6061 aluminum alloy under all pH conditions. Under pH 4.8 conditions, the acidic environment accelerated the dissolution of the aluminum alloy surface protective film, resulting in the greatest corrosion weight loss. At pH 6.8, the surface film was relatively stable and the corrosion weight loss was the lowest. Under pH 8.8 conditions, aluminum and its oxides/hydroxides may undergo amphoteric dissolution, weakening the protective function of the film layer and causing a slight increase in corrosion weight loss.

### 3.2. Corrosion Morphology and Product Analysis

**Figure 2:**
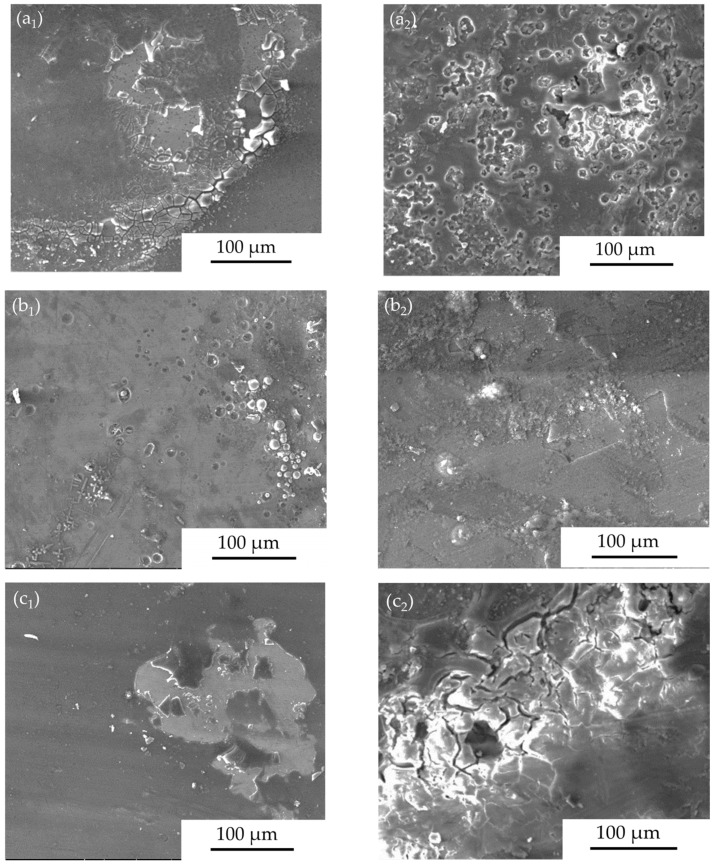
SEM surface morphologies of 6061 aluminum alloy after immersion in propylene glycol coolant with different pH values for 14 days: (**a_1_**,**b_1_**,**c_1_**) uncoupled condition at pH 4.8, 6.8 and 8.8, respectively; (**a_2_**,**b_2_**,**c_2_**) galvanically coupled with 304 stainless steel at pH 4.8, 6.8 and 8.8, respectively.

Figure 2 shows the microstructural corrosion morphology of the anode material (6061 aluminum alloy) in a galvanic couple and the 6061 aluminum alloy in a single-metal state after 14 days of immersion in propylene glycol coolant at different pH levels. As shown in Figure 2, the surface morphology of the 6061 aluminum alloy in the single-metal state changes significantly with pH. At pH = 4.8, the specimen surface exhibited significant accumulation of corrosion products and localized corrosion pits, indicating that the acidic environment promoted the dissolution and destruction of the aluminum alloy’s surface oxide film. Since HCl was used to adjust the coolant to acidic conditions, the introduced Cl^−^ also contributed to the corrosion process. Cl^−^ could adsorb on defects or weak sites of the oxide/hydroxide film and penetrate the film, thereby promoting local film breakdown and pit initiation. Therefore, the severe localized corrosion observed at pH 4.8 can be attributed to the combined effects of H^+^-induced film dissolution and Cl^−^-induced local film damage. In the SEM image at the 100 μm scale, the uncoupled specimen at pH 4.8 showed localized corroded regions with a lateral size of approximately 100–200 μm, accompanied by cracked corrosion products and scattered pit-like defects. This morphology indicates that the surface oxide/hydroxide film was unstable under acidic conditions, and local film breakdown exposed the aluminum substrate to further dissolution. At pH = 6.8, the specimen surface was relatively smooth with few corrosion products, showing only a small number of scattered corrosion defects. This suggests that the near-neutral environment favored the maintenance of a relatively continuous protective film on 6061 aluminum alloy, thereby suppressing localized film rupture and pit initiation. When the pH rose to 8.8, localized corrosion products and areas of film damage reappeared on the surface. Different from the acidic condition, the damage at pH 8.8 was mainly characterized by localized product accumulation, film cracking, and partial peeling, with the damaged region reaching a lateral scale of approximately 100 μm. This morphology is consistent with the amphoteric dissolution behavior of aluminum oxides/hydroxides in weakly alkaline media: OH^−^ can react with Al(OH)_3_ or Al_2_O_3_ to form soluble Al(OH)_4_^−^, which weakens the integrity of the protective film and causes local cracking or peeling. At pH 8.8, the uncoupled specimen mainly exhibited localized film peeling and product accumulation, and the damaged region reached a lateral scale of approximately 100 μm. Compared to the single-metal state, the surface corrosion characteristics of 6061 aluminum alloy were more pronounced after galvanic coupling. Under pH = 4.8 conditions, the surface of the coupled specimens exhibited denser corrosion pits and uneven corrosion products, indicating that galvanic action in an acidic environment further accelerated the anodic dissolution of the 6061 aluminum alloy. For the coupled specimen at pH 4.8, more than several tens of pit-like defects were observed within the selected SEM field, and the characteristic diameter of most pits was in the range of several micrometers to about 20 μm. The presence of Cl^−^ further enhanced the susceptibility of 6061 aluminum alloy to localized corrosion by weakening the compactness of the surface film, while galvanic coupling accelerated the anodic dissolution process. As a result, the coupled specimen at pH 4.8 exhibited higher pit density and more uneven corrosion products than the uncoupled specimen. This result indicates that, after the acidic medium damaged the surface film, galvanic coupling with 304 stainless steel further promoted electron transfer from 6061 aluminum alloy to 304 stainless steel, thereby accelerating local anodic dissolution and increasing the density of corrosion defects. Under pH = 6.8 conditions, the surface of the coupled specimens remained relatively smooth with relatively mild corrosion, suggesting that a near-neutral environment can mitigate galvanic corrosion to some extent. Under pH = 8.8 conditions, the surface of the coupled specimens exhibited obvious localized corrosion product coverage, as well as cracking or peeling of the protective film. For the coupled specimen at pH 8.8, the cracked and peeled corrosion-product-covered region extended over a lateral scale greater than 200 μm in the SEM observation field. This indicates that the weakly alkaline dissolution of the aluminum oxide/hydroxide film and the galvanic acceleration of anodic dissolution acted together, leading to more evident localized film destabilization than that observed in the uncoupled specimen. This indicates that the stability of the aluminum alloy surface film decreases in a weakly alkaline environment, and the galvanic coupling effect further promotes the development of localized corrosion.

**Figure 3:**
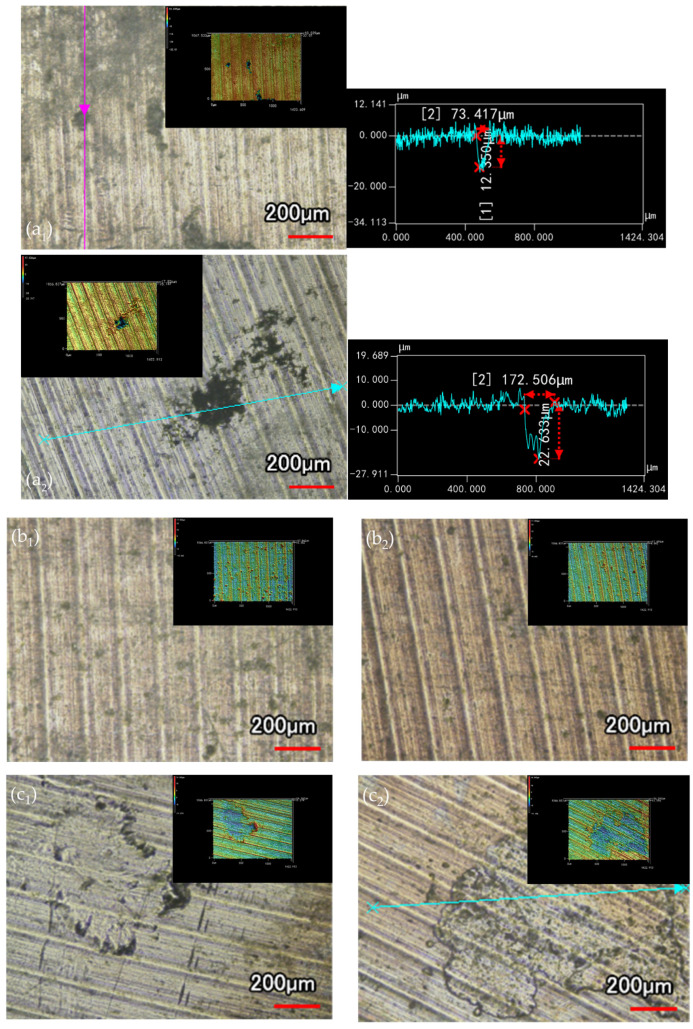
3D laser confocal morphologies of 6061 aluminum alloy after immersion in propylene glycol coolant with different pH values for 14 days and removal of corrosion products: (**a_1_**,**b_1_**,**c_1_**) uncoupled condition at pH 4.8, 6.8 and 8.8, respectively; (**a_2_**,**b_2_**,**c_2_**) galvanically coupled with 304 stainless steel at pH 4.8, 6.8 and 8.8, respectively.

Figure 3 shows the 3D confocal morphologies of the 6061 aluminum alloy anode material in a galvanic couple and the single-metal 6061 aluminum alloy after removal of corrosion products in propylene glycol coolant at different pH levels. When the propylene glycol coolant pH was 4.8, multiple localized corrosion pits were observed on the surface of 6061 aluminum alloy. In the selected 3D confocal observation regions, the characteristic depth and lateral size of representative pits on the uncoupled 6061 aluminum alloy were approximately 12.35 μm and 73.42 μm, respectively. After galvanic coupling with 304 stainless steel, the corresponding characteristic pit depth and lateral size increased to approximately 22.63 μm and 172.51 μm, respectively. Compared with the uncoupled specimen, the pit depth and width increased by about 83.3% and 135.0%, respectively. These representative morphological measurements indicate that galvanic coupling intensified localized pit propagation rather than representing an isolated corrosion feature. Galvanic coupling not only increased the number of localized defects observed by SEM, but also promoted the inward growth and lateral expansion of corrosion pits under acidic conditions. This pit propagation behavior may also be associated with Cl^−^ introduced during pH adjustment with HCl. Once local defects formed on the aluminum oxide/hydroxide film, Cl^−^ could accumulate within the occluded pit regions and hinder the repassivation of the exposed aluminum substrate, thereby sustaining localized dissolution. Under galvanic coupling, the anodic polarization of 6061 aluminum alloy further intensified this process, resulting in more obvious pit deepening and lateral enlargement at pH 4.8. In propylene glycol coolant with a pH of 6.8, no significant corrosion pits were observed on the surfaces of either the coupled or uncoupled 6061 aluminum alloys. When the pH of the propylene glycol coolant was 8.8, extensive spalling occurred in localized areas on the surface of the 6061 aluminum alloy in both states. The damaged regions at pH 8.8 were mainly irregular peeling or spalling areas rather than single measurable pits, and their lateral size reached the scale of more than 200 μm in the selected confocal images. This indicates that the dominant damage mode under weakly alkaline conditions was not typical pit deepening, but local film dissolution, cracking, and detachment caused by the formation of soluble Al(OH)_4_^−^. This indicates that under slightly alkaline conditions, although aluminum alloys typically possess a certain degree of passivation capability, excessively high pH may cause localized destabilization or dissolution of the surface film, leading to a resurgence of localized corrosion.

**Figure 4:**
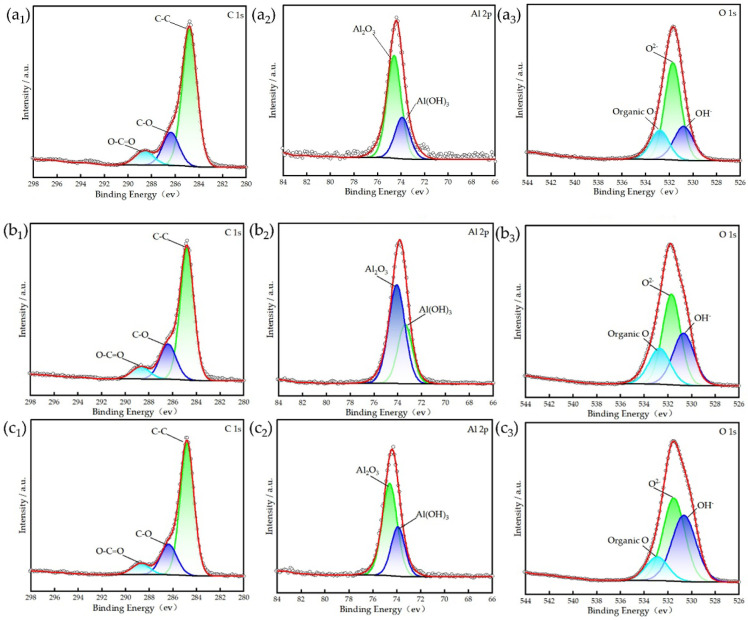
XPS spectra of 6061 aluminum alloy after immersion in propylene glycol coolant with different pH values for 14 days: (**a_1_**–**a_3_**) pH 4.8; (**b_1_**–**b_3_**) pH 6.8; (**c_1_**–**c_3_**) pH 8.8.

**Figure 5:**
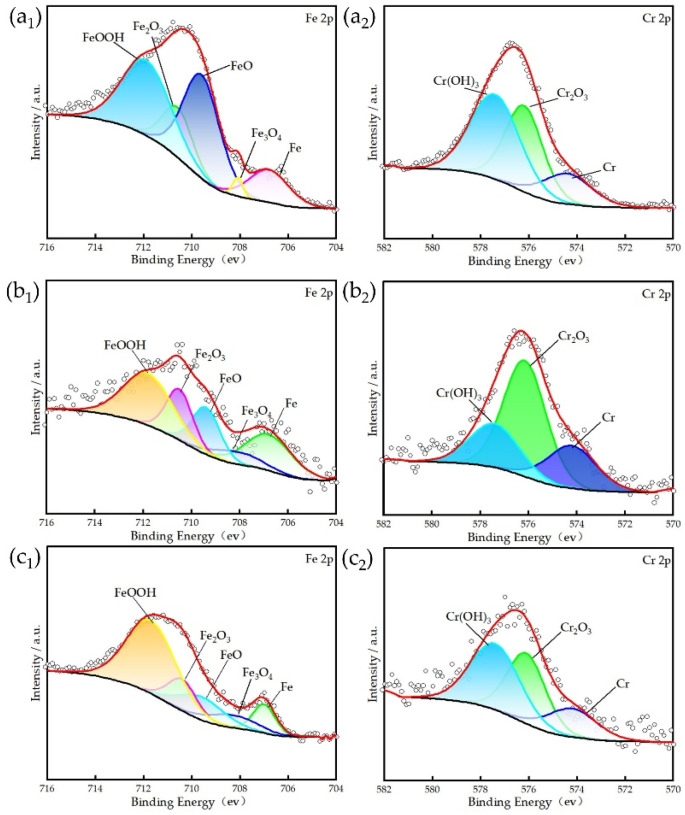
XPS spectra of 304 stainless steel after immersion in propylene glycol coolant with different pH values for 14 days: (**a_1_**–**a_3_**) pH 4.8; (**b_1_**–**b_3_**) pH 6.8; (**c_1_**–**c_3_**) pH 8.8.

**Figure 6:**
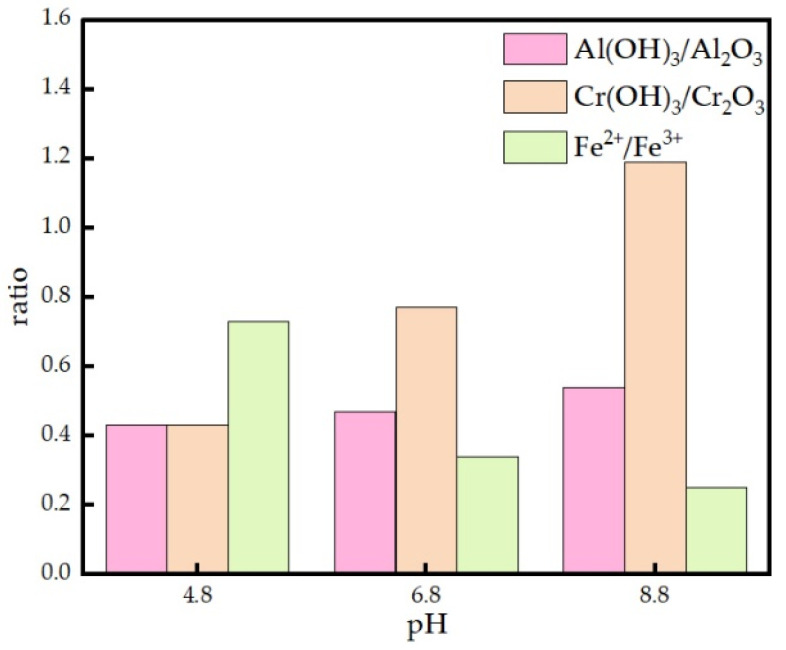
Proportions of different species in XPS spectra under different pH conditions.

Figure 4 and Figure 5, as well as Table 2 and Figure 6, present the X-ray photoelectron spectroscopy (XPS) spectra and the composition of various substances for 6061 aluminum alloy and 304 stainless steel after being immersed in propylene glycol coolant for 14 days at different pH values. As shown in Figure 4, the main components of the corrosion products of 6061 aluminum alloy in propylene glycol coolant at different pH levels remained unchanged, consisting primarily of Al(OH)_3_ and Al_2_O_3_. Under different pH conditions, the C 1s spectrum of the 6061 aluminum alloy exhibited three peaks, corresponding to C–C, C–O, and O–C=O bonds, respectively. The C–O component is mainly related to hydroxyl-containing propylene glycol species adsorbed on the aluminum surface, while the O–C=O component may originate from oxidized glycol-derived organic species formed during immersion. These oxygen-containing organic groups can interact with surface Al–OH or Al^3+^ sites through hydrogen bonding or coordination, resulting in the formation of an organic-containing interfacial layer on the Al(OH)_3_/Al_2_O_3_ corrosion product film. Such PG-derived adsorption species may reduce the direct contact between the aluminum substrate and water/oxygen-containing electrolyte, thereby contributing to the inhibition of anodic dissolution. However, under acidic or weakly alkaline conditions, dissolution or destabilization of the aluminum oxide/hydroxide film weakens this adsorption-related protection, leading to increased localized corrosion. Combined with the organic oxygen observed in the O 1s spectrum, it can be concluded that propylene glycol is adsorbed on the sample surface. Figure 5 and Figure 6 show that the main composition of the passivation film on the surface of 304 stainless steel remains largely consistent under different pH conditions, consisting primarily of Fe and Cr oxides and hydroxides; however, the relative content of each component changes with pH. At pH = 4.8, the Cr(OH)_3_/Cr_2_O_3_ ratio is the lowest, which may be due to the faster dehydroxylation reaction in acidic solutions [47]; Furthermore, when the propylene glycol coolant has a pH of 4.8, the Fe^2+^/Fe^3+^ ratio is highest. This is likely because, at lower pH values, the dissolution rate of Fe^3+^ is faster, and Fe in the inner substrate is oxidized to lower-valent oxides [48].

### 3.3. Electrochemical Analysis of Corrosion

**Figure 7:**
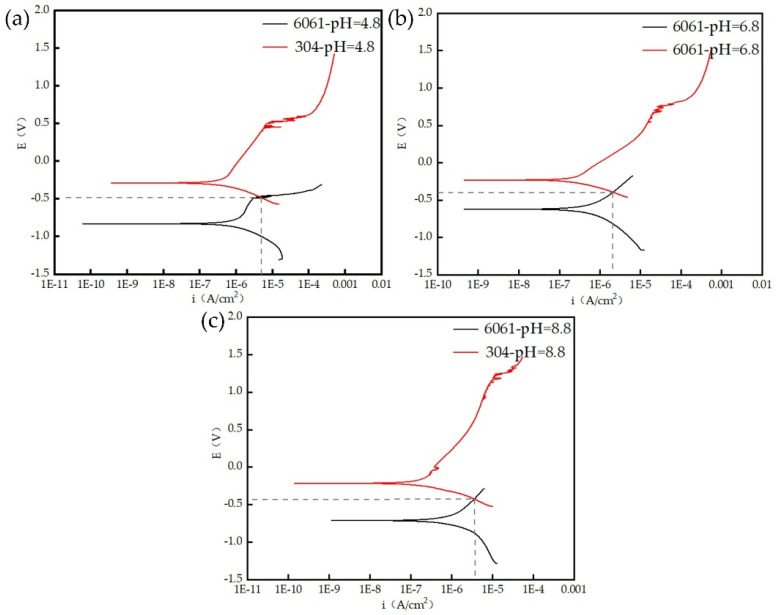
Polarization curves of 6061 aluminum alloy and 304 stainless steel in propylene glycol coolant with different pH values and the changes induced by galvanic corrosion: (**a**) pH 4.8; (**b**) pH 6.8; (**c**) pH 8.8.

Figure 7 and Table 3 show the polarization curves and fitting results for 6061 aluminum alloy and 304 stainless steel in propylene glycol coolant at pH 4.8, pH 6.8, and pH 8.8, respectively. When the pH of the propylene glycol coolant is 4.8, the corrosion potentials of 6061 aluminum alloy and 304 stainless steel are −834 mV and −289 mV, respectively. The significant potential difference between the two materials results in a pronounced galvanic effect, with a corresponding coupled current density of 4933 nA/cm^2^. In addition, the acidic environment was adjusted using HCl, and the introduced Cl^−^ can adsorb on defects in the oxide/passive film and promote local film breakdown. Therefore, the combined effect of H^+^ and Cl^−^ further weakens the surface film stability of the metals, especially 6061 aluminum alloy, resulting in a higher corrosion current density and stronger galvanic corrosion tendency at pH 4.8. When the pH of the propylene glycol coolant increased to 6.8, the potential difference between the two materials decreased relatively, and the corresponding coupling current density also decreased to 2581 nA/cm^2^. When the pH of the propylene glycol coolant was increased to 8.8, the corrosion potentials of 6061 aluminum alloy and 304 stainless steel were −717 mV and −217 mV, respectively; the potential difference increased again, leading to an enhanced galvanic corrosion effect. The mixed potential corresponding to the intersection of the polarization curves was −425 mV, and the coupled current density reached 3651 nA/cm^2^.

**Figure 8:**
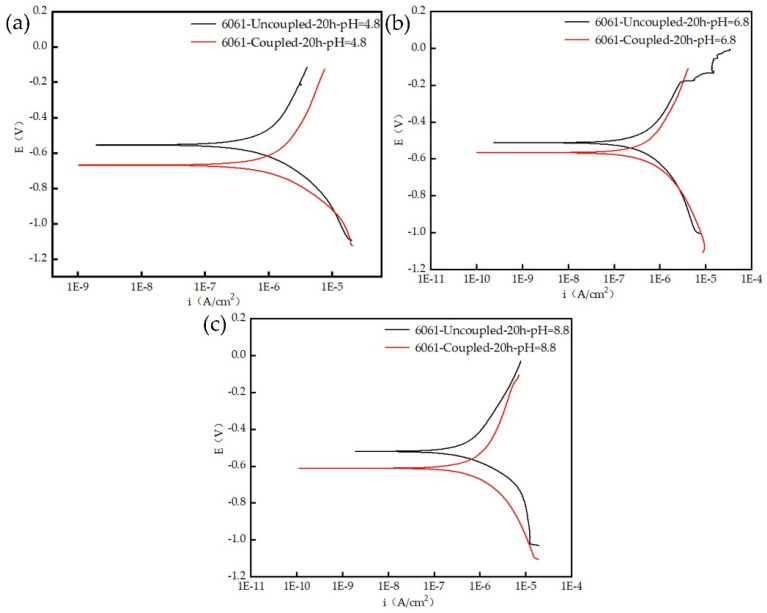
Polarization curves of 6061 aluminum alloy after immersion in propylene glycol coolant with different pH values for 20 h under coupled and uncoupled conditions. (**a**) pH 4.8; (**b**) pH 6.8; (**c**) pH 8.8.

**Figure 9:**
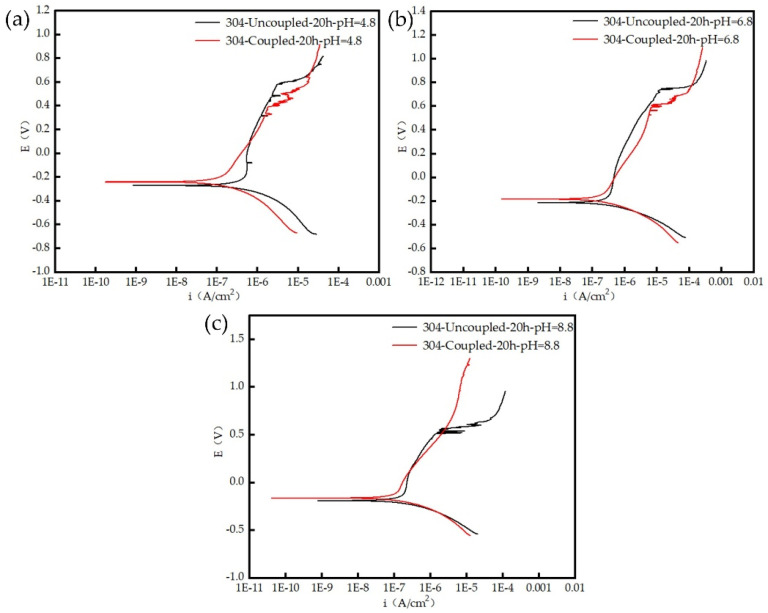
Polarization curves of 304 stainless steel after immersion in propylene glycol coolant with different pH values for 20 h under coupled and uncoupled conditions. (**a**) pH 4.8; (**b**) pH 6.8; (**c**) pH 8.8.

Figure 8 and Figure 9, along with Table 4 and Table 5, show the polarization curves and fitting results for 6061 aluminum alloy and 304 stainless steel after 20 h of immersion in propylene glycol coolant at different pH levels, under both coupled and uncoupled conditions. The corrosion current density of 6061 aluminum alloy increased after coupling under different pH conditions, indicating that the galvanic effect between the two materials significantly accelerated the corrosion of 6061 aluminum alloy after coupling. This galvanic effect was most pronounced under acidic conditions and weakest under near-neutral conditions. Furthermore, the corrosion current density of 304 stainless steel after coupling remained consistently low, indicating that 304 stainless steel acts as a cathode in the galvanic system, thereby inhibiting corrosion and maintaining a stable passivated state.

**Figure 10:**
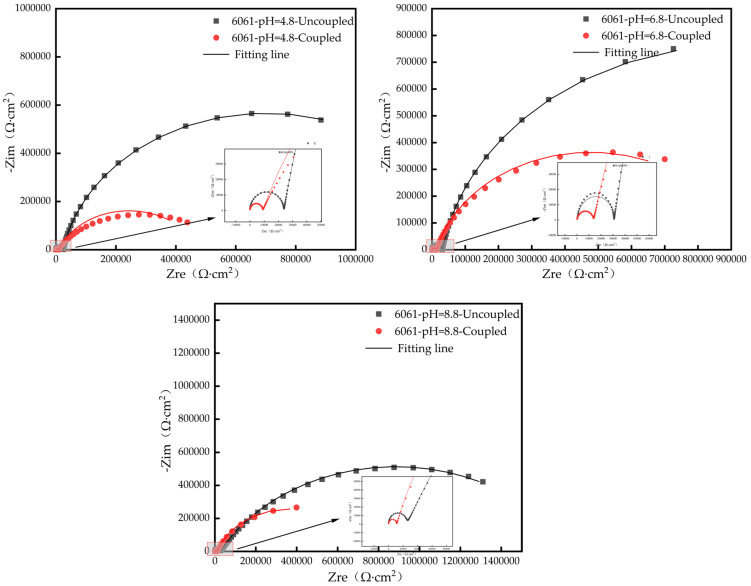
Electrochemical impedance spectra of 6061 aluminum alloy after immersion in propylene glycol coolant with different pH values for 20 h under uncoupled and galvanically coupled conditions: (**a**) pH 4.8; (**b**) pH 6.8; (**c**) pH 8.8.

**Figure 11:**
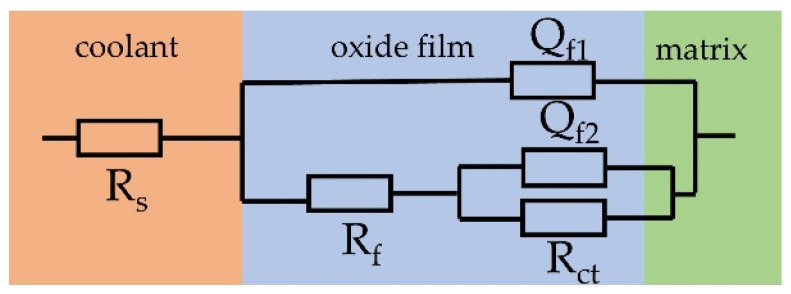
Equivalent circuit models for 6061 aluminum alloy in propylene glycol coolant.

Figure 10 shows a schematic diagram of the electrochemical impedance of 6061 aluminum alloy after 20 h of immersion in propylene glycol coolant at different pH levels, under both coupled and uncoupled conditions. In the Nyquist plots, the diameter of the capacitive arc can be used to qualitatively evaluate the corrosion resistance of the electrode surface, while the low-frequency impedance modulus in the Bode plots reflects the overall barrier performance of the surface film and the charge-transfer process. A larger capacitive arc and a higher low-frequency impedance generally indicate a more stable oxide film and a lower corrosion rate. The electrochemical impedance of the 6061 aluminum alloy was fitted using the equivalent circuit shown in Figure 11, and the fitting results are shown in Table 6. The equivalent circuit contains two time constants, which correspond to the response of the surface oxide film and the charge-transfer process at the metal/film interface, respectively. Here, Rs represents the resistance of the propylene glycol coolant, Rf represents the resistance of the oxide film on the 6061 aluminum alloy, and R_ct_ represents the charge transfer resistance at the surface of the 6061 aluminum alloy matrix. Due to the presence of a certain “diffusion effect,” the constant-phase element Q_f1_ represents the interfacial capacitance of the metal-oxide film, and the constant-phase element Q_f2_ represents the interfacial capacitance at the surface of the metal matrix. Furthermore, the commonly used polarization resistance R_p_ represents the resistance of the system during electrochemical reactions, and polarization resistance R_p_ = R_f_ + R_ct_. The smaller the value of R_p_, the higher the degree of corrosion in the system and the greater the corrosion rate. As shown in Figure 10 and Table 6, in propylene glycol coolants with different pH values, both the oxide film resistance R_f_ and the polarization resistance R_p_ of 6061 aluminum alloy in the coupled state are smaller than those in the uncoupled state. This result is consistent with the smaller capacitive arc radius and lower impedance modulus observed in the coupled state, indicating that galvanic coupling weakens the protective effect of the oxide film and accelerates the anodic dissolution of 6061 aluminum alloy. This indicates that 6061 aluminum alloy consistently acts as the anode in propylene glycol coolants at pH 4.8, 6.8, and 8.8, with electrons flowing from 6061 aluminum alloy to 304 stainless steel. Furthermore, when the propylene glycol coolant pH is 4.8, the polarization resistance (R_p_) of the 6061 aluminum alloy in the coupled state decreases by approximately 65% compared to that in the uncoupled state; when the propylene glycol coolant pH is 6.8, the difference in polarization resistance (R_p_) between the two states of the 6061 aluminum alloy is less than 50%. This may be attributed to the combined effect of H^+^ and Cl^−^ introduced by HCl during pH adjustment. In acidic media, H^+^ promotes the dissolution of the aluminum oxide/hydroxide film, while Cl^−^ can adsorb on the defective sites of the oxide film and penetrate the film, destroying its integrity and promoting localized corrosion. The synergistic action of H^+^ and Cl^−^ therefore significantly decreases the stability and protective ability of the oxide film on 6061 aluminum alloy. By contrast, under near-neutral conditions, the passive film on 6061 aluminum alloy is relatively stable, which reduces the difference in impedance response between the coupled and uncoupled states. When the pH increases to 8.8, partial dissolution of the aluminum oxide/hydroxide film may occur in the alkaline medium, resulting in a certain decrease in film stability compared with the near-neutral condition. Therefore, the EIS results further confirm that the galvanic corrosion of 6061 aluminum alloy is most severe in the acidic environment, while it is relatively alleviated near pH 6.8.

**Figure 12:**
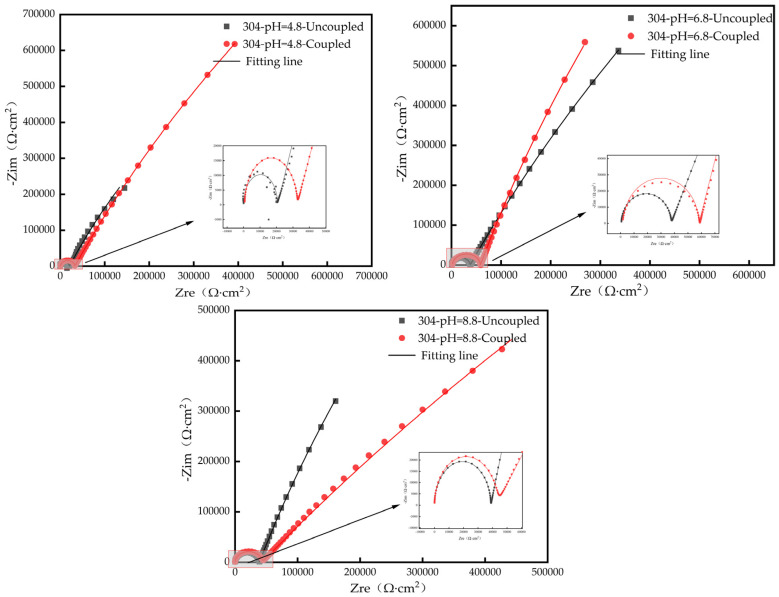
Electrochemical impedance spectra of 304 stainless steel after immersion in propylene glycol coolant with different pH values for 20 h under uncoupled and galvanically coupled conditions: (**a**) pH 4.8; (**b**) pH 6.8; (**c**) pH 8.8.

**Figure 13:**
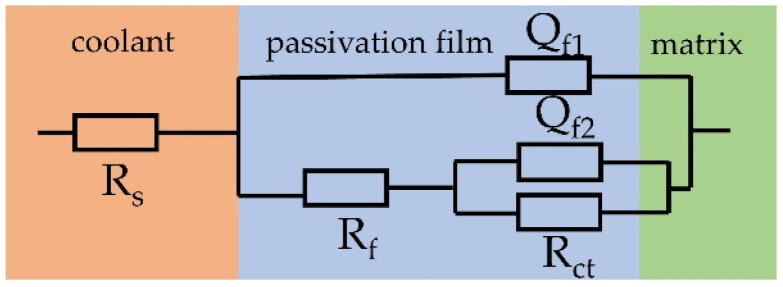
Equivalent circuit models for 304 stainless steel in propylene glycol coolant.

Figure 12 shows a schematic diagram of the electrochemical impedance of 304 stainless steel after 20 h of immersion in propylene glycol coolant at different pH levels, under both coupled and uncoupled conditions. The Nyquist plots of 304 stainless steel show typical capacitive behavior, and the Bode plots further reveal the impedance response associated with the passive film and interfacial charge transfer. The higher low-frequency impedance modulus indicates better protection of the passive film and a lower corrosion tendency. The electrochemical impedance of 304 stainless steel was fitted using the equivalent circuit shown in Figure 13, and the fitting results are shown in Table 7. Similar to 6061 aluminum alloy, the equivalent circuit with two time constants was used to describe the electrochemical response of the passive film and the metal/passive-film interface. Figure 12 and Table 7 demonstrate that the electrochemical impedance of 304 stainless steel varies under different pH conditions. In the uncoupled state, as the pH increased from 4.8 to 6.8, the polarization resistance R_p_ of 304 stainless steel increased; when the pH further increased to 8.8, the polarization resistance R_p_ increased further, indicating that the passivation film on the surface of 304 stainless steel is more stable under near-neutral and weakly alkaline conditions; R_p_ was minimal under acidic conditions, indicating that 304 stainless steel has a stronger tendency to corrode at pH = 4.8. This is because the H^+^ in acidic media can promote the dissolution of iron oxides/hydroxides, while Cl^−^ introduced by HCl can attack the Cr-rich passive film and promote local film breakdown. The presence of Cl^−^ weakens the compactness and stability of the passive film, thereby reducing the impedance response of 304 stainless steel under acidic conditions. Under the same pH conditions, both the oxidation film resistance (R_f_) and the charge transfer resistance (R_ct_) of 304 stainless steel in the coupled state were greater than those in the uncoupled state. The increase in R_f_ indicates that the passive film on 304 stainless steel becomes more protective after coupling, while the increase in R_ct_ suggests that the anodic dissolution reaction on the stainless-steel surface is suppressed. This indicates that when 304 stainless steel forms a galvanic couple with 6061 aluminum alloy, it primarily acts as the cathode, and its anodic dissolution process is suppressed, resulting in a milder degree of surface corrosion. Meanwhile, the cathodic reaction on 304 stainless steel, mainly oxygen reduction, promotes electron consumption on its surface and helps maintain the stability of the passive film. Therefore, although Cl^−^ in the acidic coolant can reduce the passive film stability of 304 stainless steel, galvanic coupling makes 304 stainless steel act mainly as the cathode, suppressing its anodic dissolution. The EIS results are therefore consistent with the galvanic corrosion mechanism: 6061 aluminum alloy acts as the anodic material and suffers accelerated corrosion, whereas 304 stainless steel acts as the cathodic material and exhibits enhanced impedance response and improved corrosion resistance after coupling.

### 3.4. Evaluation of Galvanic Corrosion Sensitivity

**Figure 14:**
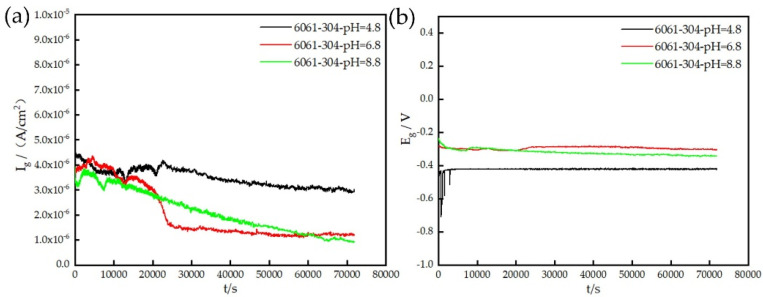
Galvanic current and galvanic potential of the 6061 aluminum alloy/304 stainless steel galvanic couple in propylene glycol coolant with different pH values: (**a**) galvanic current; (**b**) galvanic potential.

Figure 14 shows a schematic diagram of the thermocouple current and potential for a 6061 aluminum alloy/304 stainless steel thermocouple pair in propylene glycol coolant at pH values of 4.8, 6.8, and 8.8. At the beginning of the test, the galvanic current of the 6061 aluminum alloy/304 stainless steel couple in propylene glycol coolant with three different pH values fluctuated to some extent, all showing a trend of first increasing and then decreasing; as the test duration increased, the galvanic current of the couple in the propylene glycol coolant tended to stabilize. This is because, when the specimens first came into contact with the coolant, the oxide film on the surface of the 6061 aluminum alloy and the passivation film on the surface of the 304 stainless steel had not yet reached a stable state, and the processes of anodic dissolution and cathodic reduction were relatively active. As the immersion time increased, a relatively stable oxide film gradually formed on the surface of the 6061, and the passivation film on the surface of the 304 also tended to stabilize, with the system gradually reaching a dynamic equilibrium. Using Equation (1) to calculate the galvanic current density of the galvanic couple in propylene glycol coolants with pH values of 4.8, 6.8, and 8.8, the results were 3.49 μA/cm^2^, 2.03 μA/cm^2^, and 2.14 μA/cm^2^, respectively. Referring to Table 1 to assess their galvanic corrosion sensitivity, the corrosion sensitivity of the galvanic couple in propylene glycol coolants at pH 6.8 and pH 8.8 is classified as Grade C, while in the propylene glycol coolant at pH 4.8, the corrosion sensitivity increases to Grade D. The test results indicate that propylene glycol coolant with a pH of 4.8 exhibits the highest corrosivity toward the 6061 aluminum alloy/304 stainless steel galvanic couple; however, the change in pH did not alter the anode and cathode roles of the couple, meaning that 6061 aluminum alloy consistently acts as the anode material and 304 stainless steel consistently acts as the cathode material in propylene glycol coolant.

As shown in Figure 14b, the galvanic potential of the galvanic couple in propylene glycol coolant at pH 6.8 and pH 8.8 eventually stabilized at −0.29 V and −0.34 V, respectively; the difference in galvanic potential between the two pH conditions was not significant. The galvanic potential of the pair in propylene glycol coolant at pH 4.8 eventually stabilized near −0.42 V. That is, the galvanic potential of the 6061 aluminum alloy and 304 stainless steel pair was lowest in propylene glycol coolant at pH 4.8, indicating the greatest tendency for galvanic corrosion between the two, which is consistent with the results of the galvanic current test.

## 4. Discussion

In an acidic propylene glycol coolant with a pH of 4.8, the oxide film on the surface of 6061 aluminum alloy undergoes acid dissolution first, as shown by the following reaction:

(2) $${\mathrm{Al}}_{2}O_{3}{+6H}^{+}\to {2\mathrm{Al}}^{3+}{+3H}_{2}O$$

(3) $$\mathrm{Al}{\left(\mathrm{OH}\right)}_{3}{+3H}^{+}\to {\mathrm{Al}}^{3+}{+3H}_{2}O$$

Once the protective film on the specimen’s surface is damaged, the exposed aluminum substrate undergoes anodic dissolution, while the cathode continues to reduce dissolved oxygen. The reaction is as follows:

(4) $$\mathrm{Al}\to {\mathrm{Al}}^{3+}{+3e}^{-}$$

(5) $$O_{2}{+2H}_{2}{O+4e}^{-}\to {4\mathrm{OH}}^{-}$$

Under pH 4.8 conditions, H^+^ continuously erodes the surface protective film, causing the aluminum matrix to dissolve continuously and exacerbating corrosion. When the specimen is coupled with 304 stainless steel, electrochemical test results show that the potential of 6061 aluminum alloy is more negative under acidic conditions; it continues to act as an anode, dissolving to form Al^3+^, while 304 stainless steel consistently acts as a cathode, undergoing an oxygen reduction reaction. Under acidic conditions, the surface film of the 6061 aluminum alloy is severely damaged by H^+^, resulting in the most pronounced anodic activation. Meanwhile, although the 304 stainless steel acts as a cathode and does not dissolve, an oxygen reduction reaction continuously occurs on its surface, consuming electrons and driving the 6061 aluminum alloy to continuously lose electrons. Consequently, both the rate of electron generation at the anode and the rate of electron consumption at the cathode are high within the system, leading to the maximum galvanic current and exacerbating the degree of corrosion.

For a weakly alkaline propylene glycol coolant with a pH of 8.8, the oxide film on the surface of 6061 aluminum alloy is no longer damaged by acidity; however, since aluminum is an amphoteric metal, the Al(OH)_3_ and Al_2_O_3_ on its surface undergo an alkaline dissolution reaction, which can be expressed as:

(6) $$\mathrm{Al}{\left(\mathrm{OH}\right)}_{3}{+\mathrm{OH}}^{-}\to {\left[\mathrm{Al}{\left(\mathrm{OH}\right)}_{4}\right]}^{-}$$

(7) $${\mathrm{Al}}_{2}O_{3}{+2\mathrm{OH}}^{-}{+3H}_{2}O\to 2{\left[\mathrm{Al}{\left(\mathrm{OH}\right)}_{4}\right]}^{-}$$

When the surface protective film dissolves, the aluminum substrate dissolves to form Al^3+^, and the cathode continues to undergo an oxygen reduction reaction to produce OH^−^. The OH^−^ generated by the cathodic reaction then combines with Al^3+^ to form a new corrosion product film. However, this film is unstable and continues to be broken down by the solution. Consequently, the surface film of 6061 aluminum alloy is constantly forming and breaking down, reducing its protective effect and increasing the degree of corrosion compared to near-neutral conditions. When coupled with 304 stainless steel, the 6061 aluminum alloy continues to dissolve as the anode, while the 304 stainless steel still undergoes the oxygen reduction reaction. Compared to pH = 6.8, under pH = 8.8 conditions, the surface film of the 6061 aluminum alloy undergoes alkaline dissolution due to the action of OH^−^, and the anode activity is re-enhanced. At the same time, the passivation film on the surface of 304 stainless steel becomes more stable, allowing its cathodic oxygen reduction reaction to proceed more continuously. Consequently, the galvanic current in the system increases again compared to pH 6.8, and galvanic corrosion intensifies.

## 5. Conclusions

This study investigates the corrosion behavior of 6061 aluminum alloy, 304 stainless steel, and their galvanic couples in propylene glycol coolants at pH 4.8, pH 6.8, and pH 8.8. The conclusions are as follows:

(1) As the pH of the propylene glycol coolant increases, the corrosion resistance of 6061 aluminum alloy first increases and then decreases. The corrosion of 6061 aluminum alloy is most severe at pH = 4.8, weakest at pH = 6.8, and increases again at pH = 8.8. Compared with 6061 aluminum alloy, 304 stainless steel shows better corrosion resistance under all tested pH conditions.
(2) XPS results indicate that as the pH of the propylene glycol coolant increases, the corrosion products of the 6061 aluminum alloy remain unchanged, consisting of Al(OH)_3_ and Al_2_O_3_; for 304 stainless steel, the main components of the passivation film remain unchanged under different pH conditions.
(3) As the pH of the propylene glycol coolant increases, the galvanic current between 6061 aluminum alloy and 304 stainless steel in the coolant first decreases and then increases, while the galvanic potential first increases and then decreases. Furthermore, 6061 aluminum alloy consistently acts as the anode and 304 stainless steel as the cathode in the galvanic couple, with no reversal of the anode and cathode occurring. For propylene glycol coolants at pH 4.8, 6.8, and 8.8, the corresponding galvanic corrosion sensitivity grades are Grade D, Grade C, and Grade C, respectively.
(4) For mixed 6061 aluminum alloy/304 stainless steel propylene glycol cooling systems, the coolant pH should be maintained in a near-neutral range. Based on the tested results, pH 6.5–7.5 is recommended as the practical control range, with pH 6.8 as the preferred target value. Coolant acidification toward pH 4.8 should be avoided, and excessive alkalization toward pH 8.8 is not recommended for aluminum-containing systems.

## 6. Limitations and Future Work

This study clarified the effect of pH on the self-corrosion and galvanic corrosion behavior of 6061 aluminum alloy/304 stainless steel in 25 vol.% propylene glycol coolant. However, some limitations remain. First, only three pH values, namely 4.8, 6.8, and 8.8, were investigated, and the optimal pH range needs to be further refined using more intermediate pH values. Second, HCl and NaOH were used for pH adjustment, which inevitably introduced Cl^−^ and Na^+^ ions and changed the ionic strength of the coolant. Therefore, the influence of different pH regulators and ion contamination should be further separated in future work. Third, the tests were conducted under static immersion and naturally aerated conditions, while practical cooling systems usually involve coolant flow, temperature fluctuation, and long-term circulation.

Future work should focus on long-term dynamic loop tests under flowing coolant conditions, combined with continuous monitoring of pH, conductivity, dissolved oxygen, and galvanic current. In addition, the effects of coolant aging, repeated thermal cycling, and inhibitor depletion should be investigated to establish more reliable coolant replacement criteria. Aluminum-compatible inhibitors, such as organic carboxylates, silicates, or their combined formulations, should also be further evaluated to optimize the corrosion protection of mixed aluminum alloy/stainless steel cooling systems.

## Figures and Tables

**Table 1:** Criteria for evaluating galvanic corrosion susceptibility.

Electrode Couple Current Density μA/cm^2^	Electrochemical Couple Corrosion Sensitivity Rating
*i_g_* ≤ 0.3	A
0.3 < *i_g_* ≤ 1.0	B
1.0 < *i_g_* ≤ 3.0	C
3.0 < *i_g_* ≤ 10.0	D
*i_g_* ≥ 10.0	E

**Table 2:** Material composition under different pH conditions.

Test Environment	Material Proportion/%							
	Al		Cr			Fe		
	Al(OH)_3_	Al_2_O_3_	Cr	Cr(OH)_3_	Cr_2_O_3_	Fe	Fe^2+^	Fe^3+^
pH = 4.8	30	70	23	23	54	12	37	51
pH = 6.8	32	68	16	34	50	6	21	73
pH = 8.8	35	65	19	44	37	10	18	72

**Table 3:** Fitting results for 6061 aluminum alloy and 304 stainless steel in propylene glycol coolant with different pH values.

Material	pH	Corrosion Potential /mV	Mixed Potential/mV	Corrosion Current Density /nA·cm^−2^	Coupling Current /nA·cm^−2^
6061	4.8	−834	−483	669	4933
	6.8	−625	−397	332	2581
	8.8	−717	−425	483	3651
304	4.8	−289	−483	190	4933
	6.8	−234	−397	132	2581
	8.8	−217	−425	105	3651

**Table 4:** Fitting results of polarization curves for 6061 aluminum alloy after immersion in propylene glycol coolant with different pH values for 20 h under coupled and uncoupled conditions.

Test Condition	pH	Corrosion Potential/mV	Corrosion Current Density /nA·cm^−2^
Uncoupled	4.8	−554	431
	6.8	−515	246
	8.8	−520	319
Coupled	4.8	−670	656
	6.8	−570	296
	8.8	−612	394

**Table 5:** Fitting results of polarization curves for 304 stainless steel after immersion in propylene glycol coolant with different pH values for 20 h under coupled and uncoupled conditions.

Test Condition	pH	Corrosion Potential/mV	Corrosion Current Density /nA·cm^−2^
Uncoupled	4.8	−280	174
	6.8	−214	130
	8.8	−200	102
Coupled	4.8	−245	106
	6.8	−185	103
	8.8	−165	80

**Table 6:** EIS fitting results of 6061 aluminum alloy soaked in propylene glycol coolant with different pH values for 20 h under non-coupled and electrically coupled conditions.

pH	Condition	R_s_/Ω·cm^2^	Q_f1_ × 10^−9^/Ω^−1^·cm^−2^·S^n^	n_1_	R_f_ × 10^3^/Ω·cm^2^	Q_f2_ × 10^−6^/Ω^−1^·cm^−2^·S^n^	n_2_	R_ct_ × 10^5^/Ω·cm^2^	R_p_ × 10^5^/Ω·cm^2^
4.8	Uncoupled	135.15	1.28	0.98	23.93	1.80	0.69	13.56	13.80
	Coupled	138.20	2.05	0.99	9.16	6.01	0.80	4.69	4.79
6.8	Uncoupled	114.53	1.15	0.97	30.99	8.69	0.90	17.84	18.15
	Coupled	168.37	8.70	0.90	13.93	6.93	0.85	9.27	9.41
8.8	Uncoupled	145.96	2.01	0.95	25.65	6.11	0.89	17.15	17.41
	Coupled	125.33	2.24	0.99	11.23	7.26	0.77	7.07	7.18

**Table 7:** EIS fitting results of 304 stainless steel soaked in propylene glycol coolant with different pH values for 20 h under non-coupled and electrically coupled conditions.

pH	Condition	R_s_/Ω·cm^2^	Q_f1_ × 10^−9^/Ω^−1^·cm^−2^·S^n^	n_1_	R_f_ × 10^3^/Ω·cm^2^	Q_f2_ × 10^−6^/Ω^−1^·cm^−2^·S^n^	n_2_	R_ct_ × 10^5^/Ω·cm^2^	R_p_ × 10^5^/Ω·cm^2^
4.8	Uncoupled	145.12	1.53	0.98	20.65	8.10	0.57	32.48	32.69
	Coupled	135.20	2.08	0.96	31.93	3.07	0.74	66.05	66.37
6.8	Uncoupled	124.21	2.16	0.98	35.67	1.00	0.73	40.92	41.28
	Coupled	136.52	1.54	0.94	36.86	2.65	0.81	69.08	69.50
8.8	Uncoupled	158.25	1.90	0.95	38.79	1.51	0.82	57.10	57.49
	Coupled	145.97	1.96	0.98	42.04	1.20	0.67	70.50	71.02

## Data Availability

The original contributions presented in this study are included in the article. Further inquiries can be directed to the corresponding author.
